# Research on the Attenuation Characteristics of High-Frequency Elastic Waves in Rock-Like Material

**DOI:** 10.3390/ma15196604

**Published:** 2022-09-23

**Authors:** Xiling Liu, Feng Xiong, Qin Xie, Xiukun Yang, Daolong Chen, Shaofeng Wang

**Affiliations:** 1 School of Resources and Safety Engineering, Central South University, Changsha 410083, China; 2 State Key Laboratory of Coal Resources and Safe Mining, China University of Mining and Technology, Xuzhou 221116, China

**Keywords:** elastic wave, frequency dependent attenuation, attenuation coefficient, rock microstructures

## Abstract

In order to study the frequency-dependent attenuation characteristics of high-frequency elastic waves in rock-like materials, we conducted high-frequency elastic wave attenuation experiments on marble, granite, and red sandstone rods, and investigated the frequency dependence of the attenuation coefficient of high-frequency elastic waves and the frequency dependence of the attenuation of specific frequency components in elastic waves. The results show that, for the whole waveform packet of the elastic wave signal, the attenuation coefficient and the elastic wave frequency have an approximate power relationship, with the exponents of this power function being 0.408, 0.420, and 0.384 for marble, granite, and red sandstone, respectively, which are close to 1/2 the exponent value obtained theoretically by the Kelvin–Voigt viscoelastic model. However, when the specific frequency components are tracked during the elastic wave propagation, the exponents of the power relationship between the attenuation coefficient and frequency are 0.982, 1.523, and 0.860 for marble, granite, and red sandstone, respectively, which indicate that the relationship between the attenuation coefficient and frequency is rock-type dependent. Through the analysis of rock microstructure, we demonstrate that this rock-type-dependent relationship is mainly caused by the scattering attenuation component due to the small wavelength of the high-frequency elastic wave. Therefore, the scattering attenuation component may need to be considered when the Kelvin–Voigt model is used to describe high-frequency elastic wave attenuation in rock-like materials. The results of this research are of good help for further understanding the attenuation characteristics of high-frequency elastic waves in rock-like materials.

## 1. Introduction

When an elastic wave propagates in a medium, attenuation will occur, which is accompanied by a decrease in energy, amplitude, and frequency with increasing distance. The causes of attenuation can be attributed to geometrical spreading, internal friction, mode conversion, and scattering during propagation [1]. Thereinto, the attenuation caused by internal friction is strongly correlated with the properties of the medium, which is the main reason for the attenuation of elastic waves.

The understanding of wave attenuation in rock media originates from the study of seismic waves. As early as 1941, Born estimated the attenuation coefficients of the earth based on seismic reflection records [2], and the quality factor Q was introduced to describe the absorption of elastic waves by the material and the inelasticity of the material, and to characterize the attenuation. Q is defined as the ratio of the total energy *E*_0_ to the energy Δ*E* consumed by the vibration in a cycle [3]. Futterman [4] first discussed the basic characteristics of seismic wave attenuation in a rock mass and pointed out that its amplitude attenuation was related to the quality factor, the velocity, frequency of seismic waves, etc. Since then, many scholars have conducted research on the attenuation characteristics in various rock masses as well as on the influences of wave velocity, frequency, temperature, pressure, fluid viscosity and permeability, and propagation type (P wave or S wave) [5,6,7,8,9]. An in-depth understanding of elastic wave attenuation will facilitate the acquisition of the source characteristics according to the data collected by the sensor; additionally, the internal properties of the rock mass may be inverted based on the understanding of attenuation [10]. Similarly, the study of the attenuation characteristics of elastic waves in small-scale rock samples may elucidate the physical properties of rocks [11,12,13,14,15,16].

In laboratory rock deformation tests, the acoustic emission (AE) technique is often used to study the failure characteristics of rocks by recording the elastic waves released during crack initiation, propagation, and penetration [17,18,19,20,21,22,23]. It is also necessary to deeply understand the attenuation characteristics of the AE signal in the rock sample [24,25,26,27] and then obtain the characteristics of the fracture source in the rock sample. As the rock AE signal is generally higher than 100 kHz [28], the attenuation of elastic waves with a high frequency is also a subject of concern. In research on the attenuation of high-frequency AE signals in rock-like materials, Dobrin et al. [29] summarized that the amplitude of the AE signal obeys the exponential attenuation law, based on a large number of laboratory experiments. Furthermore, as the medium through which elastic waves propagate in a rock-like material is viscoelastic, the viscoelasticity of the medium is often studied, and many viscoelastic medium theories have been proposed [30,31]. 

As the attenuation is frequency dependent, many frequency-dependent attenuation theories have also been proposed. Müller believed that in rocks containing pore fluids, the squirting flow mechanism and wave-induced flow also affect the attenuation of elastic waves, which is frequency dependent [32]. The viscosity effect is also frequency dependent, and considering the viscoelasticity of the rock mass, the attenuation caused by internal friction is also frequency dependent [33,34]. The continuous development and progress of measurement technology have resulted in more accurate attenuation characteristic parameters being obtained to explore the frequency dependence of attenuation [35]. However, a high-frequency elastic wave signal attenuates rapidly, which makes the attenuation measurement of such elastic wave more difficult. Therefore, there have been many studies focusing on the attenuation of elastic waves at the seismic exploration frequency, but the attenuation of high-frequency elastic waves is not fully understood.

**Figure 1:**
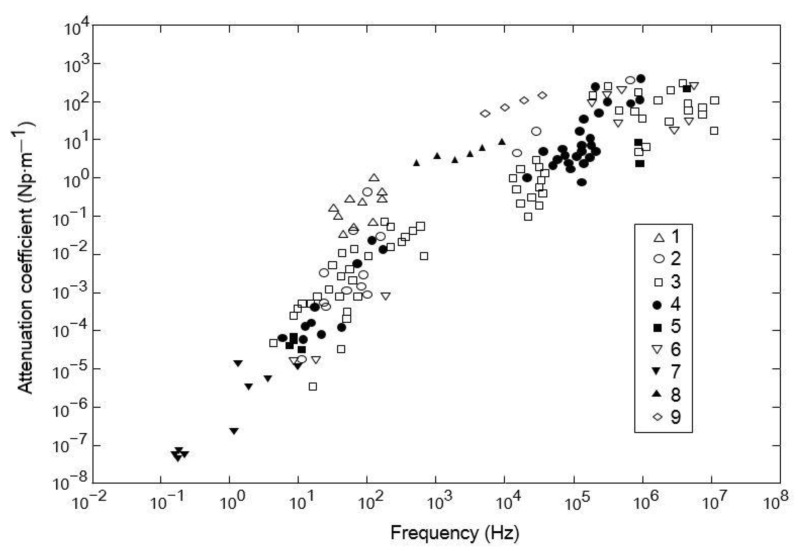
Relationship between the attenuation coefficients of various rocks and frequency: 1—uncemented sedimentary rock; 2—semi-cemented sedimentary rock; 3—solidified sedimentary rock; 4,5—magmatic rock; 6: metamorphic rock; 7—results of deep seismic reflections; 8—limestone; 9—sandstone (dry).

Figure 1 shows the distribution of various rock attenuation coefficients at different frequencies, which was obtained by summarizing a large amount of experimental data by Schön et al. [36]. In the low-frequency range, the attenuation coefficient increases approximately linearly with the increasing of frequency, while in the high-frequency range (higher than 10^5^ Hz), the attenuation coefficients of the various rock types are not well correlated with the frequency. Furthermore, in research on the attenuation characteristics in rocks, small samples are analyzed according to the characteristics of the signal collected by the sensor after the elastic wave is generated by the seismic source and passes through the sample; however, such an experimental design cannot fully describe the detailed attenuation process in the propagation path of elastic waves, and the acquisition of the attenuation process in the propagation path would greatly facilitate the understanding of the propagation characteristics of high-frequency signals in rock samples.

In this study, we researched the frequency dependence of attenuation in the frequency range of 100 kHz–1 MHz. By conducting attenuation experiments on elastic waves with different frequencies and using rock rods of a different lithology, we determined the attenuation coefficients (α) of elastic waves in rocks. We then analyzed the relationship between α and *f* based on the Kelvin–Voigt viscoelasticity theory. Moreover, we explained the differences in attenuation velocity and frequency dependence in different lithological rocks by examining the microstructure of the rocks. Furthermore, we explored the relationship between the attenuation coefficient and frequency of dry rocks in the high-frequency range, and discussed the attenuation process during the propagation of elastic waves in rocks. The results of this study will facilitate an understanding of the frequency-dependent characteristics of attenuation and also provide a theoretical basis for inverting the source characteristics with the signal collected by the sensor in rock AE tests.

## 2. Frequency-Dependent Theories of *α*

In wave science, there are many theories characterizing wave propagation [6,37,38,39]. During wave propagation, the medium will cause a loss in wave energy, and the attenuation caused by the absorption and scattering accounts for a large part of the total attenuation. Scattering is significant when the wavelength is close to the particle size of the medium. The absorption attenuation of dry rock is mainly the friction dissipation caused by the relative motion of the grain boundary and the crack surface and the energy consumption caused by the residual strain. The absorption attenuation in a rock medium is often related to the viscoelasticity of the rock and is frequency dependent. Absorption attenuation in rock media can be described using a viscoelastic medium model. In viscoelastic media, the stress–strain relationship is no longer a simple linear relationship and many assumptions have been put forward. Different incomplete elastic medium models have been established. The Maxwell and Kelvin–Voigt models are classic.

The Maxwell model comprises an elastic element in series with a viscous element. When stress is applied to the Maxwell model, it is applied equally on the two components, and the strain includes two parts: an elastic body and a viscous body. Owing to the series connection of the elements, the strain in the model is transient. For a stress *σ*_0_, the strain *ε* of the model is expressed as follows:

(1) $$\varepsilon =\frac{\sigma_{0}}{E}+\frac{\sigma_{0}}{\eta }t$$

where *E* is the elastic modulus, *η* is the viscosity coefficient of the viscous element, and *t* is time. According to Equation (1), as long as a tiny force acts on the Maxwell body, the strain will increase infinitely, and when a tiny stress is unloaded, the model will exhibit irreversible viscous deformation, which is inconsistent with the actual characteristics of the rock mass disturbed by elastic waves. 

**Figure 2:**
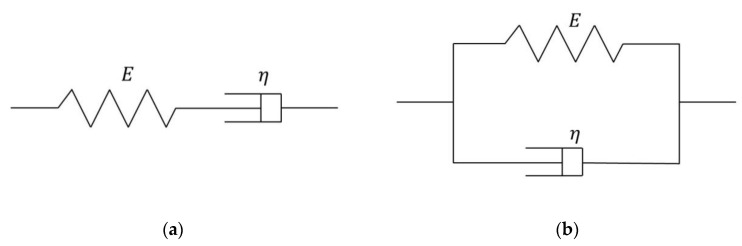
Classic viscoelastic medium models: (**a**) Maxwell model; (**b**) Kelvin–Voigt model.

The elements contained in the Kelvin–Voigt model are the same as those in the Maxwell model (Figure 2), except that the elastic and viscous elements are connected parallelly. When stress is applied, the strains of the two elements are the same, and the total stress is the sum of the two stresses. When the Kelvin–Voigt model is subjected to a stress of *σ*_0_,

(2) $$\sigma_{0}=E\varepsilon +\eta \frac{d\varepsilon }{dt}$$

this ordinary differential equation should be solved:

(3) $$\varepsilon =\frac{\sigma_{0}}{E} [1-exp\left(-\frac{E}{\eta }t\right)]$$

The strain of the Kelvin–Voigt body is not instantaneous after being stressed, and it also shows the hysteresis characteristics of strain after stress disappears, which can better reflect the stress–strain relationship of the internal particles, owing to the viscoelastic characteristics of the rock material under the action of elastic waves. Kelvin–Voigt bodies are commonly used in seismic exploration. Based on this model, some scholars have used numerical calculations to simulate the propagation law of seismic wave fields in viscoelastic media [40,41]. 

The Kelvin–Voigt model is brought into the elastic equation of space:

(4) $$\left.\begin{array}{c} \sigma_{x}=\lambda \theta +2\mu \frac{\partial u}{\partial x}+\lambda^{\prime }\frac{\partial \theta }{\partial t}+2\mu^{\prime }\frac{\partial }{\partial t}\frac{\partial u}{\partial x}\\ \sigma_{y}=\lambda \theta +2\mu \frac{\partial v}{\partial y}+\lambda^{\prime }\frac{\partial \theta }{\partial t}+2\mu^{\prime }\frac{\partial }{\partial t}\frac{\partial v}{\partial y}\\ \sigma_{z}=\lambda \theta +2\mu \frac{\partial w}{\partial z}+\lambda^{\prime }\frac{\partial \theta }{\partial t}+2\mu^{\prime }\frac{\partial }{\partial t}\frac{\partial w}{\partial z}\\ \tau_{xy}=\mu \left(\frac{\partial v}{\partial x}+\frac{\partial u}{\partial y}\right)+\mu^{\prime }\frac{\partial }{\partial t}\left(\frac{\partial v}{\partial x}+\frac{\partial u}{\partial y}\right)\\ \tau_{yz}=\mu \left(\frac{\partial w}{\partial y}+\frac{\partial v}{\partial z}\right)+\mu^{\prime }\frac{\partial }{\partial t}\left(\frac{\partial w}{\partial y}+\frac{\partial v}{\partial z}\right)\\ \tau_{zx}=\mu \left(\frac{\partial u}{\partial z}+\frac{\partial w}{\partial x}\right)+\mu^{\prime }\frac{\partial }{\partial t}\left(\frac{\partial u}{\partial z}+\frac{\partial w}{\partial x}\right) \end{array}\right\}$$

where $\sigma_{x},\sigma_{y},\;\;\mathrm{and}\;\sigma_{z}$ are the normal stress components in the *x*-, *y*-, and *z*-axes, respectively; $\tau_{xy}$, $\tau_{yz},$ and $\tau_{zx}$ are the shear stress components in the *x*-, *y*-, and *z*-axes, respectively; *u*, *v*, and *w* are the displacement components in the *x*-, *y*-, and *z*-axes, respectively; λ and μ are the Lamé coefficients; and $\lambda^{\prime }\;\mathrm{and}\;\mu^{\prime }$ are the viscosity coefficients that characterize the viscous properties of the medium.

The equilibrium equation can be obtained from Equation (4), regardless of the body force:

(5) $$\left.\begin{array}{c} \rho \frac{\partial^{2}u}{\partial t^{2}}=\left(\lambda +\mu \right)\frac{\partial \theta }{\partial x}+\mu \nabla^{2}u+\left(\lambda^{\prime }+\mu^{\prime }\right)\frac{\partial }{\partial t}\frac{\partial \theta }{\partial x}+\mu^{\prime }\frac{\partial }{\partial t}\nabla^{2}u\\ \rho \frac{\partial^{2}v}{\partial t^{2}}=\left(\lambda +\mu \right)\frac{\partial \theta }{\partial y}+\mu \nabla^{2}v+\left(\lambda^{\prime }+\mu^{\prime }\right)\frac{\partial }{\partial t}\frac{\partial \theta }{\partial y}+\mu^{\prime }\frac{\partial }{\partial t}\nabla^{2}v\\ \rho \frac{\partial^{2}w}{\partial t^{2}}=\left(\lambda +\mu \right)\frac{\partial \theta }{\partial z}+\mu \nabla^{2}w+\left(\lambda^{\prime }+\mu^{\prime }\right)\frac{\partial }{\partial t}\frac{\partial \theta }{\partial z}+\mu^{\prime }\frac{\partial }{\partial t}\nabla^{2}w \end{array}\right\}$$

where ρ is the density of the medium, θ is the volume strain, and $\nabla^{2}$ means $\frac{\partial^{2}}{\partial x^{2}}+\frac{\partial^{2}}{\partial y^{2}}+\frac{\partial^{2}}{\partial z^{2}}$.

This is the differential equation of motion in a viscoelastic body based on the Kelvin–Voigt model, regardless of the body force. It describes the absorption of waves by the medium through the first-order partial derivatives of the two displacements with respect to time.

Consider a plane harmonic P-wave propagating along the positive x-direction, which only contains the u component in the x-direction and is independent of the y- and z-directions of the components. Owing to the irrotational characteristics of the P-wave, we can simplify Equation (5) and obtain the wave equation of the P-wave in a viscoelastic medium:

(6) $$\rho \frac{\partial^{2}u}{\partial t^{2}}=\left(\lambda +2\mu \right)\frac{\partial^{2}u}{\partial x^{2}}+\left(\lambda^{\prime }+2\mu^{\prime }\right)\frac{\partial^{2}}{\partial x^{2}}\frac{\partial u}{\partial t}$$

Then we can write the displacement equation of the P wave:

(7) $$u\left(x,t\right)=Ae^{-\alpha x}e^{-i\left(\omega t-kx\right)}$$

where ω is the angular frequency, k is the wave number, and i is the imaginary number. By substituting Equation (7) into Equation (6) and making the real part equal to the imaginary part, the expression of the attenuation coefficient α can be obtained:

(8) $$\alpha ={ [\frac{\rho \beta \omega^{2}}{2\left(\beta^{2}+{\beta^{\prime }}^{2}\omega^{2}\right)}\left(\sqrt{1+\frac{{\beta^{\prime }}^{2}\omega^{2}}{\beta^{2}}}-1\right)]}^{1/2}$$

where $\beta =\lambda +2\mu ,\;\beta \prime =\lambda^{\prime }+2\mu^{\prime }$, and β and $\beta^{\prime }$ are constant. Equation (7) shows that the attenuation coefficient α is a function of frequency; that is, the attenuation of elastic waves at different frequencies in a viscoelastic medium is different. When the frequency of the wave is high,

$$\frac{{\beta^{\prime }}^{2}\omega^{2}}{\beta^{2}}\gg 1$$

Additionally, ω=2πf, so Equation (8) can be approximated as

(9) $$\alpha \approx {\left(\frac{\rho \pi f}{\beta^{\prime }}\right)}^{1/2}$$

According to Equation (9), the attenuation coefficient α is proportional to the square root of the frequency f. When the frequency increases, the attenuation coefficient increases as well. With the propagation of the elastic wave, high-frequency components are quickly absorbed.

## 3. Experimental Setup

**Figure 3:**
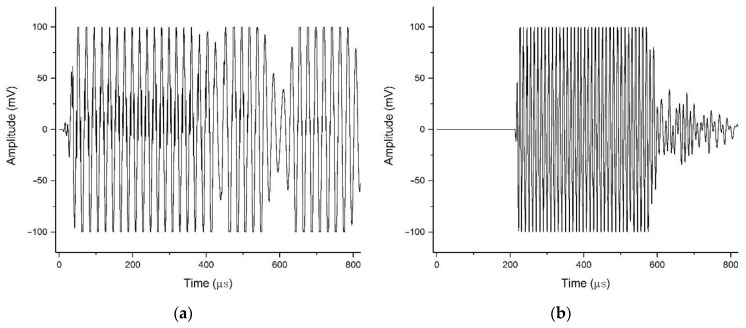
The waveform of the generated signal in (**a**) 50 kHz and (**b**) 100 kHz.

The samples used in the experiment were long rectangular rods consisting of three different types of rocks (marble, granite, and red sandstone). Relevant information on the samples is presented in Table 1. Ultrasonic transducers (ϕ=25 mm) were used to generate elastic waves at six different frequencies (50 kHz, 100 kHz, 250 kHz, 500 kHz, 750 kHz, and 1 MHz, and the generated elastic waves are of the sine type, as shown in Figure 3, taking 50 kHz and 100 kHz excitations as examples). Vallen AMSY-6 AE equipment with 32 channels was used as the acquisition device. The signals were collected by a VS45-H-type wide-frequency response piezoelectric sensor with a resonant frequency of 20 kHz to 450 kHz. The preamplifier gain, threshold value, and sampling rate of the signal acquisition system were set to 34 dB, 35 dB, and 10 MHz, respectively.

**Figure 4:**
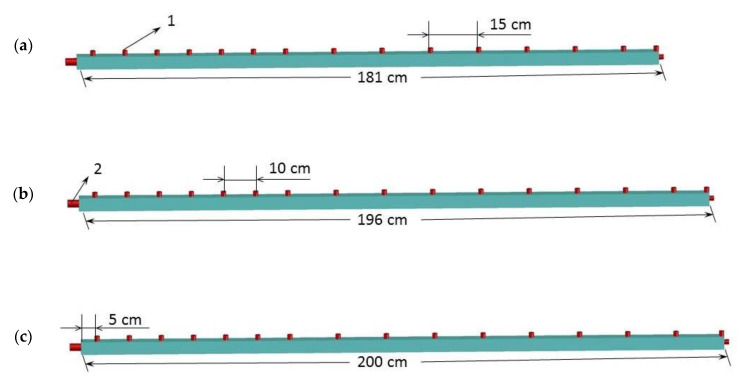
Sensor arrangement for (**a**) red sandstone, (**b**) marble, and (**c**) granite: 1—sensors for elastic wave collection; 2—transducer for elastic wave excitation with various frequencies.

In the test, the transducer was placed at the end of the rock rod. To maintain the position of the transducer in the center of the rock rod end face, suitable cushion blocks were selected to hold the rock rod during the test. To explore the attenuation changes of elastic waves with different frequencies over distance, signal collection sensors were arranged at different locations on one side of the rock rod, and another signal collection sensor was placed at the other rock rod end face opposite to the one where the excitation transducer was fixed. The deployment of the elastic wave excitation transducer and the signal collection sensor is shown in Figure 4 and Table 2. The coupling agent was applied between the sensor, transducer, and contact surface of the rock, and the sensor was fixed with a magnetic suction fixture to prevent it from falling off during the test. After all sensors were pasted, their sensitivity was calibrated individually using lead break tests.

## 4. Results

### 4.1. Attenuation Characteristics of the Excitation Sources with Different Frequencies

**Figure 5:**
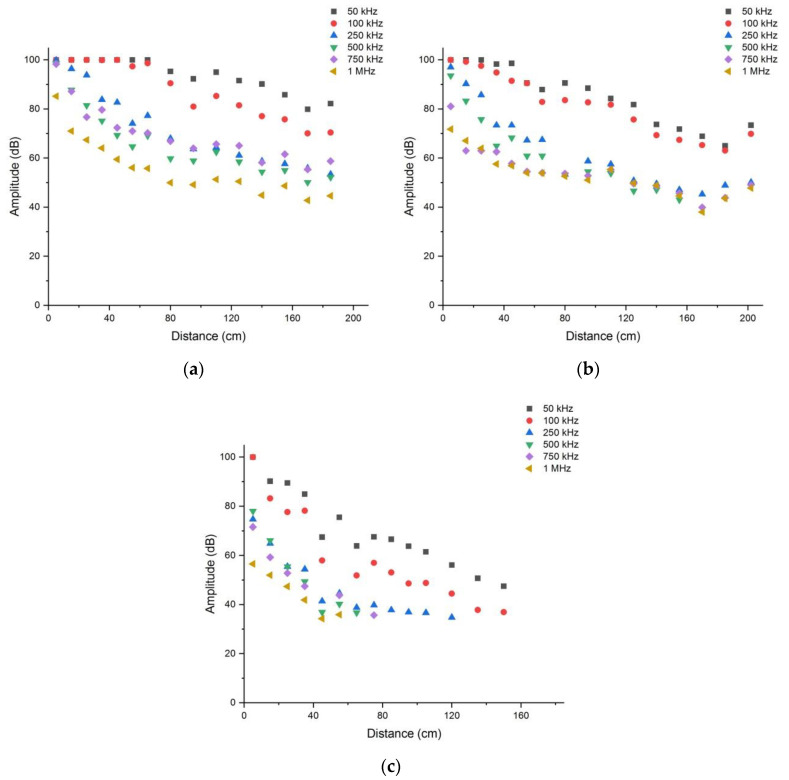
Attenuation of amplitudes in (**a**) marble, (**b**) granite, and (**c**) red sandstone at different frequencies.

Figure 5 shows the attenuation of the amplitude with distance for marble, granite, and red sandstone at different frequencies. Owing to the upper limit of the amplitude collected by the sensor, the amplitudes at the first several AE sensors on the marble and granite samples were all 100 dB. The amplitude of the wave in the red sandstone was significantly attenuated, and the sensors located at the back could not receive valid waveform information. As the frequency increased, the amplitude collected by the sensor at the same distance decreased. The decreasing speed of the curve was also accelerated with an increase in frequency, and the attenuation was accelerated. Additionally, the attenuation coefficient of the rock sample increased with an increase in frequency. There were also differences in attenuation between the different rock samples. Based on the inclination of the curve, the attenuation in red sandstone was the highest and the attenuation in marble was the lowest.

The attenuation coefficient in the rock sample can be calculated according to the amplitude distribution obtained in the test, and the value of the attenuation coefficient can be obtained using Equation (10).

(10) $$A\left(x\right)=A_{0}exp\left(-\alpha x\right)$$

where $A_{0}$ is the amplitude at the source, α is the attenuation coefficient, x is the distance, and $A\left(x\right)$ is the amplitude of the wave propagating to x from the source. The unit of amplitude should be the voltage amplitude (mV), which can be obtained from the waveform data collected by the sensor. The calculated attenuation coefficients of different rocks at different frequencies are listed in Table 3.

**Figure 6:**
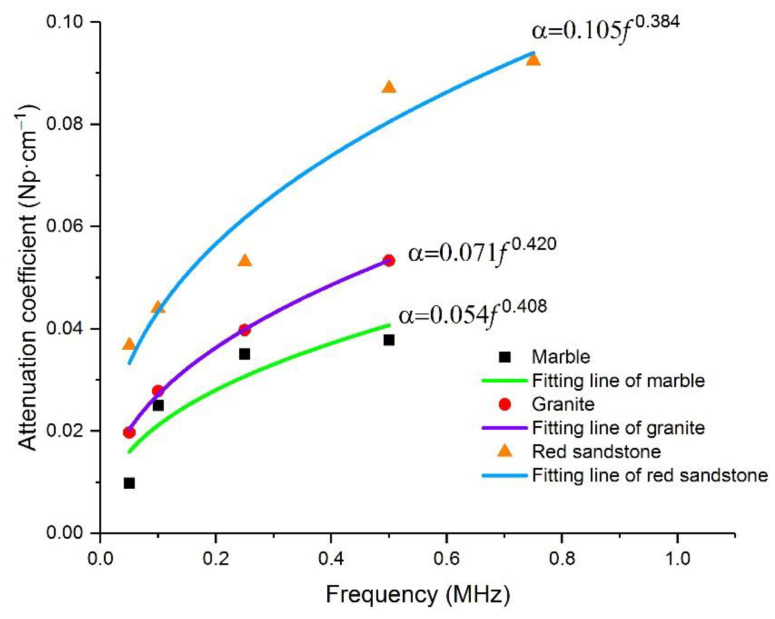
Measured attenuation coefficients of red sandstone, granite, and marble at different frequencies, and the fitted curves. It is obvious that the relationship between α and f has a similar form.

As is shown in Table 3, the attenuation coefficients of the three types of rocks increased significantly with an increase in frequency. At the initial stage of the frequency increase, the increase in attenuation coefficients was roughly proportional. However, when the frequency exceeded 500 kHz, the attenuation coefficient did not increase significantly at 750 kHz and 1 MHz. This may be related to the response frequency range of the broadband sensor for waveform collection. The resonant frequency of the signal collection sensor used in the test was 20–450 kHz; thus, the response effect of the elastic wave signal with a higher frequency may be poor. Based on the distribution characteristics of the attenuation coefficient at different frequencies (hereinafter, we use f to represent the frequency of the generated elastic wave), we fitted the correlation between α and f. The attenuation data obtained when the response was poor at frequencies of 750 kHz and 1 MHz were omitted during the fitting. The fitting results are presented in Figure 6 and Table 4.

By fitting the correlation between α and f, the attenuation coefficients in marble, granite, and red sandstone showed a power function relationship with frequency, with the exponent of the power function being very similar (0.408, 0.420, and 0.384, respectively). The value of $R^{2}$ is greater than 0.8, and the fitting degree of the granite and red sandstone fitting curves is greater than 0.9. However, the coefficients before f are different (0.105, 0.071, and 0.054 for red sandstone, granite, and marble, respectively), which lead to highest amplitude attenuation for red sandstone, followed by granite and marble. For the whole elastic wave energy packet, the attenuation mechanism of the medium due to internal friction is approximately the same. The relationship between α and f has the same formalization, but the extent of the attenuation is different due to the different composition of the medium. As a matter of fact, the effects as hysteresis and viscoelastic damping persist throughout the whole propagation process, with energy dissipation accumulating and intensifying, resulting in an exponential increase in attenuation over distance.

### 4.2. Attenuation Characteristics of Elastic Waves with Specific Frequency Components

**Figure 7:**
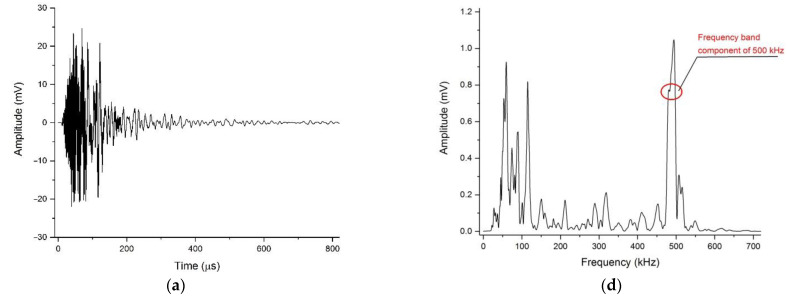

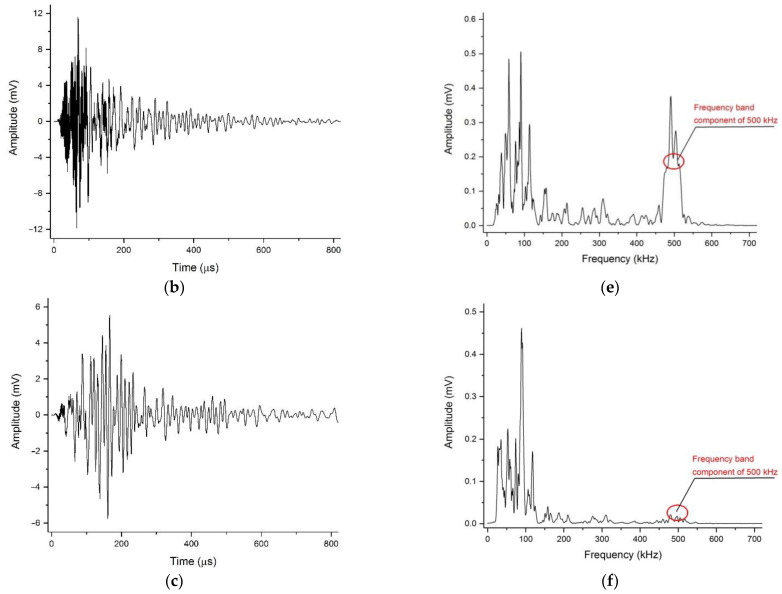
Waveforms (**a**–**c**) and amplitude spectra (**d**–**f**) at 15 cm, 25 cm, and 35 cm in marble at a frequency of 500 kHz. The red circles represent the 500 kHz components.

When an elastic wave propagates in the rock, a dispersion phenomenon occurs. The different frequency components of the wave were separated owing to the different phase velocities, and the different components of the wave had different attenuation extents. In the analysis in Section 4.1, the change in amplitude with distance reflects the attenuation of elastic waves excited with a specific frequency as a whole in the time domain, and the amplitudes used to calculate the attenuation coefficient are the maximum voltage of the whole waveform packet. In fact, as the elastic wave propagates in rock rods, the high-frequency components of the signal are gradually filtered out, but we just treat the signal of the different frequency components as a whole energy packet to investigate its attenuation characteristics, and the change in specific frequency components of the collected signal in different distances cannot be known. In this section, we will focus on the attenuation of specific frequency components in the waveform during the propagation process. In attenuation tests at various excitation frequencies, we can track the excited frequency component by performing fast Fourier transform (FFT) on the collected time-domain waveforms at different locations. Taking the 500 kHz excitation frequency attenuation test on marble as an example, as shown in Figure 7, the peak frequency (frequency with maximum amplitude in amplitude spectrum) moved from approximately 500 kHz to approximately 100 kHz, and the amplitude of the 500 kHz frequency component gradually decreases with the propagation of the elastic wave. By tracking the specific frequency component in attenuation tests at various excitation frequencies, the amplitude change of the same frequency component with distance can be obtained, thereby allowing us to analyze the attenuation characteristics of the specific frequency component.

**Figure 8:**
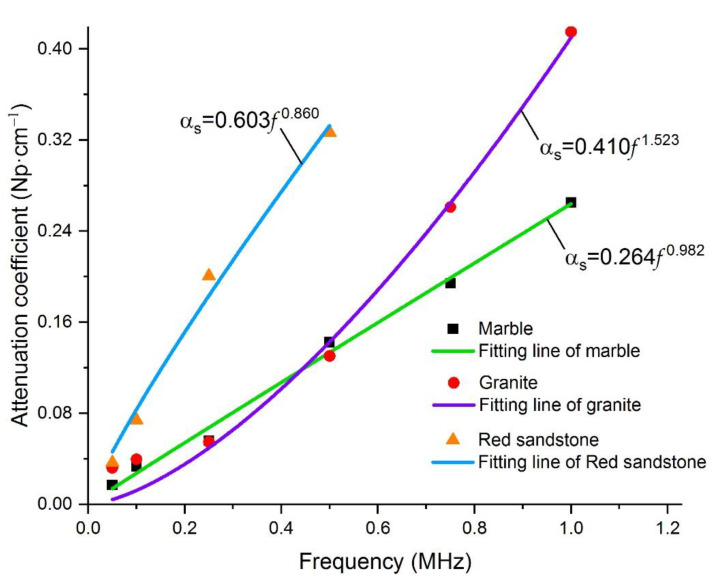
Relationship between $\alpha_{s}$ of the specific frequency components and f.

As shown in Table 5, the attenuation coefficient (here, we denote this attenuation coefficient as $\alpha_{s}$, which is calculated by tracking a specific frequency component at various distances) increases gradually with the increase in frequency, showing a similar trend in three kinds of rock rods, and the attenuation coefficient $\alpha_{s}$ and f also follow a power function relationship (as shown in Table 6 and Figure 8). However, the exponents of the power functions between $\alpha_{s}$ and f of the three kinds of rock rods are 0.982, 1.523, and 0.860 for marble, granite, and red sandstone, respectively, which vary a lot and is quite different with the results in Section 4.1, where the exponents of the power functions between α and f are almost the same. This indicates that the exponent of the power function between the attenuation coefficient and frequency is rock-type dependent; we will further discuss this phenomenon in the following section.

## 5. Discussion

Through the analysis of the experimental data, we obtained the frequency dependence of the attenuation coefficient, reflecting the amplitude of elastic waves in different rocks. In the analysis of the Kelvin–Voigt model, as shown in Equation (9), the exponent of the power function between α and f approximately equals 1/2, and the coefficient before f is determined by the density of the medium and its viscous characteristics. In Section 4.1, the exponents of the power function between α and f of the attenuation tests on marble, granite, and red sandstone are shown to be 0.408, 0.420, and 0.384, respectively, which are very close to that 1/2 theoretical value, while the coefficient before f has a lot to do with the rock type. The influence of the differences between different lithological rocks seems only to involve the coefficient of f and do not affect the exponent of power function between α and f; this is unified in the theoretical analyzing model and the experiment. This demonstrates that the Kelvin–Voigt model can describe well the correlation between the attenuation coefficient and frequency in high-frequency waves and provides a theoretical explanation for the obtained experimental results. 

**Figure 9:**
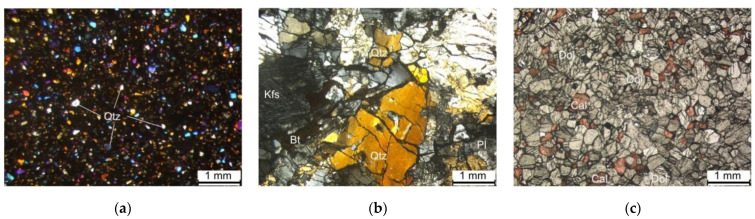
Microstructure of (**a**) red sandstone, (**b**) granite, and (**c**) marble obtained from the transparent refractive index experiments. The particle size and distribution of red sandstone and marble are relatively uniform but vary greatly in granite.

However, it should be noted that the amplitudes used to calculate the attenuation coefficient in Section 4.1 are the maximum voltage of the whole waveform packet, and the change in amplitude with distance reflects the attenuation of elastic waves as a whole energy packet. In Section 4.2, we also tracked the specific frequency component of the waveform in the attenuation tests at various excitation frequencies; the results show that the attenuation coefficient $\alpha_{s}$ of a specific frequency and f also follow a power function relationship, but the exponents of the power functions between $\alpha_{s}$ and f of the three kinds of rock rods were 0.982, 1.523, and 0.860 for marble, granite, and red sandstone, respectively, which vary a lot and is quite different to the results in Section 4.1. This indicates that the exponent of the power function between the attenuation coefficient and frequency is rock-type dependent. To further investigate this phenomenon, we obtained the microstructures of several samples through refractive index experiments of transparent sheets (Figure 9). The density of the red sandstone is the lowest; the mineral particles were small, and the gap between the particles was large. It is easy to produce relative sliding when disturbed, such that the elastic energy is converted into thermal energy dissipation, resulting in the absorption of elastic waves. The mineral particles in marble are also small, but closely combined; therefore, the elastic wave is less absorbed when propagating in the medium. This is also beneficial for reducing the attenuation caused by the reflection and refraction of waves at the particle boundary. The mineral particles in the granite sample are larger and the integrity of the particles is good; however, the internal structure was relatively broken, and there were many long-shaped gaps between the particles. This microstructure provides favorable conditions for frictional sliding between particles and increases the attenuation of elastic waves in the medium. This may also be the reason the attenuation in granite was greater than that in marble of a similar density.

Joints and fissures often exist in rock materials, and voids exist between mineral particles. These discontinuities provide favorable conditions for the sliding of adjacent fissures or particle surfaces when elastic waves pass through the medium [42]. When the elastic wave propagates in the rock material, it causes a change in the particle stress and strain, and the normal stress between the arriving interfaces increases, resulting in an increase in friction and energy loss caused by relative motion. This slippage continues until the wave passes through the interface. At this point, the direction of the frictional shear stress changes, the cracks or pores return to the original equilibrium position, and friction work is generated again. As the frequency of the wave increases, the reciprocating cycle of this friction force accelerates, resulting in a faster attenuation velocity. Therefore, the main factor causing elastic wave attenuation is the internal friction. Internal friction and scattering are medium-related factors affecting elastic wave attenuation. Separation between frictional attenuation and scattering is challenging. Matsushima et al. [43] proposed a technique to separate the two attenuations under the assumption of linear dependence of frictional attenuation on frequency, and only vertical seismic profiling (VSP) data. However, attenuation due to scattering becomes significant when the wavelength of the elastic wave is comparable to the diameter of the mineral particle. It can be seen from Figure 9 that the microstructure of red sandstone and marble exhibit similar characteristics—a uniform small particle size and distribution—while the microstructure of granite, which has a large particle size, has obvious differences with the above two kinds of rocks. When the frequency of the excited elastic wave is larger than 500 kHz, the wavelength of these high-frequency elastic waves is equivalent to the size of the mineral particles in granite, and the scattering attenuation will be significant, resulting in a higher attenuation coefficient compared to marble (the $\alpha_{s}$ of granite becomes larger than that of marble when the excited elastic wave frequency exceeds 500 kHz, as seen in Figure 8). As the mineral particles in red sandstone and marble are small, the wavelength of the excited frequency elastic waves in this test is far less comparable with the diameter of the mineral particles in red sandstone and marble; correspondingly, the scattering attenuation is not significant in these two kinds of rocks. This is also the reason why the exponent of the power functions between the $\alpha_{s}$ and f of granite is different from marble and red sandstone.

## 6. Conclusions

Elastic waves attenuate during propagation inside a rock and their amplitude and energy decrease with increasing distance. The attenuation velocities of the elastic waves with different frequencies were different in this study. Based on the experiments conducted, if the elastic waves are treated as a whole energy packet to conduct the attenuation test in rock materials, the experimental results are in good agreement with the viscoelasticity assumption based on the Kelvin–Voigt model, which deduced that the exponent of the power function relationship between the attenuation coefficient and frequency is about 1/2 and independent of rock type. However, if the specific frequency components of the elastic wave are tracked in the attenuation tests, the exponent of the power function relationship between the attenuation coefficient and frequency is rock-type dependent, which does not match the result based on the Kelvin–Voigt model. As a matter of fact, the component of scattering attenuation is not reflected in the Kelvin–Voigt model, and it cannot be reflected in the attenuation analysis of elastic waves as a whole energy packet. The results of this paper show that the attenuation analysis by tracking specific frequency components can well demonstrate the detailed causes of attenuation, and the attenuation characteristics can also be well related with the internal structure of rock material. Therefore, attention should be paid to the scattering attenuation component in high-frequency elastic wave attenuation tests in rock-like materials.

Furthermore, high-frequency signal attenuation testing requires targeted collection sensors; that is, the resonant frequency of the signal collection sensors affects the results of this type of research. Among the attenuation coefficients of the different rock samples at different frequencies, as shown in Table 3, α did not increase significantly when the frequency was higher than 500 kHz, which may be related to the poor response of the selected sensor to frequencies above 500 kHz. On the one hand, if a broadband sensor is selected, the response to the different frequency signals is not good. When selecting a narrowband sensor, the response to a certain frequency signal is good, but the response to other frequency components is poor. Based on the experimental results, it is worthwhile to consider sensors with different resonant frequencies at different locations.

## Figures and Tables

**Table 1:** Densities and dimensions of the rock samples.

Sample	Length/cm	Width/cm	Height/cm	Density/g·cm^−3^
Granite	200	4	4	2.62
Marble	196	4	4	2.60
Red sandstone	181	4	4	2.35

**Table 2:** Signal collection sensor layout scheme.

Sample	Distance (cm)											
	1	2	…	7	8	9	10	11	12	…	15	16
Granite	5	15	…	65	80	95	110	125	140	…	185	200
Marble	5	15	…	65	80	95	110	125	140	…	185	196
Red sandstone	5	15	…	65	75	85	95	105	120	…	165	181

**Table 3:** Attenuation coefficients of the samples (Np·cm^−1^).

Frequency	Marble	Granite	Red Sandstone
50 kHz	0.0098	0.0197	0.0367
100 kHz	0.0250	0.0278	0.0440
250 kHz	0.0351	0.0398	0.0531
500 kHz	0.0378	0.0533	0.0870
750 kHz	0.0342	0.0421	0.0925
1 MHz	0.0344	0.0295	0.0564

**Table 4:** Fitting relationship between α and f.

	Marble	Granite	Red Sandstone
Relationship	$\alpha =0.054f^{0.408}$	$\alpha =0.071f^{0.420}$	$\alpha =0.105f^{0.384}$
$R^{2}$	0.833	0.998	0.949

**Table 5:** Attenuation coefficients of the specific frequency components (Np·cm^−1^).

Frequency	Attenuation Coefficients $\alpha_{s}$ (Np·cm^−1^)		
	Marble	Granite	Red Sandstone
50 kHz	0.0171	0.0321	0.0368
100 kHz	0.0334	0.0397	0.0742
250 kHz	0.0560	0.0550	0.2008
500 kHz	0.1420	0.1301	0.3267
750 kHz	0.1940	0.2612	—
1 MHz	0.2655	0.4151	—

Notes: $\alpha_{s}$ is also calculated through Equation (10), with the amplitudes obtained by tracking a specific frequency component at various distances.

**Table 6:** Fitting relationship between $\alpha_{s}$ and f of the frequency components in waves.

	Marble	Granite	Red Sandstone
Relationship	$\alpha_{s}=0.264f^{0.982}$	$\alpha_{s}=0.410f^{1.523}$	$\alpha_{s}=0.603f^{0.860}$
$R^{2}$	0.994	0.985	0.990

## Data Availability

The origin date of attenuation test of elastic wave in rock rods has been uploaded to a public repository called figshare (https://figshare.com/articles/book/amplitude_and_attenuation_coefficient_xlsx/19213035) on 22 February 2022, and it is also available by contacting the corresponding author at sf.wang@csu.edu.cn.
